# Supplementary material for: Follicular Thyroid Adenoma and Follicular Thyroid Carcinoma—A Common or Distinct Background? Loss of Heterozygosity in Comprehensive Microarray Study
Source: Cancers (Basel). 2023 Jan 19;15(3):638. doi: 10.3390/cancers15030638 (PMC9913827; doi:10.3390/cancers15030638)
Supplement: Supplementary file 1 [file cancers-15-00638-s001.zip › cancers-2109814-supplementary.pdf]

Table S1. Significant genes located in the LOHs typical for both follicular thyroid carcinoma and follicular thyroid adenoma

Gene	Description	References
ATP2A1 (SERCA1)	ATPase Sarcoplasmic/Endoplasmic Reticulum Ca2+ Transporting 1; this gene encodes intracellular pump located in the sarcoplasmic or endoplasmic reticulum of the human muscle cells. It catalyses the reaction of hydrolysis of ATP, the same time coupled with the translocation of calcium ions from the cytosol to the reticulum lumen; it is involved in muscular contraction. Mutations in ATP2A1 cause some autosomal recessive forms of Brody disease, which is characterised by increasing destruction of muscular relaxation phase during exercise. It has been shown that thyroid hormones accelerate muscle relaxation by T_3 direct stimulation of *ATP2A1* (*SERCA1A)* and *ATP2A2* (*SERCA2A*) expression. Interestingly, it is known to be expressed in head and neck cancers abundantly, but also in thyroid cancer and prostate and testis.	Odermatt et al. [51]<br>Protein Atlas [52]<br>Bloise F et al. [53]<br>GeneCards database [54]<br>Hartong et al. [55]<br>Muller et al. [56]<br>Simonides et. al [57]<br>Dang et. al [58]
IL27	Encoding interleukin 27, which forms a heterodimeric complex that drives rapid expansion of naive T cells, but not memory CD4+ T cells. It also strongly synergies with interleukin 12 to trigger IFNγ production of naive CD4+ T cells. It has been shown that decreased serum level of IL27 as well as single-nucleotide polymorphisms in the IL27 gene are associated with autoimmune thyroid diseases, including Graves' disease and Hashimoto's thyroiditis among Chinese Han population. Moreover, it has been claimed that IL-27 interleukin has potent antitumor effects, the contribution of single nucleotide polymorphisms (SNPs) in IL-27 gene have been linked with the risk of papillary thyroid carcinoma, although other SNPs in IL27 gene are negatively associated with lymph node metastasis in patients with papillary thyroid carcinoma. Other research show that IL-27 level has been implicated in promoting cancer progression triggering proliferation of human leukemic cell lines. It is also known that IL-27 can be secreted by several tumour cell types, and high IL-27 levels are associated with advanced disease.	GeneCards database [54]<br>He et al. [59]<br>Saeed et al. [60]<br>Nie et al. [61]<br>Xi et al. [62]<br>Jia et al. [63]<br>Larousserie et al. [64]<br>Gonin et al. [65]<br>Kourko et al. [66]
TGFB1	This gene encodes a ligand of the TGFβ (transforming growth factor beta) protein superfamily. It is involved in regulation of the cell proliferation, differentiation, and growth, but also can modulate expression and activation of several other growth factors including IFNγ and TNFα. It is frequently upregulated in tumour cells. Finally, mutations in TGFB1 gene result in Camurati-Engelmann disease, but also several immunodeficiencies and other human diseases. In case of the thyroid function, cells of the thyroid gland express the TGFB1 gene mRNA and synthesizes the protein, which regulates thyroid growth and function, and the expression itself may be stimulated by iodine.	GeneCards database [54]<br>Pisarev et al. [67]<br>Kardalas et al. [68]<br>Mincione et al. [69]<br>Grubeck-Loebenstein et al. [70]<br>Wikipathways [71]

	It is probably produced mainly by thyroid follicular cells. It has been demonstrated that TGFβ inhibits cell proliferation. In thyroid cancer types, the inhibitory abilities of TGFβ on cell proliferation is progressively lost with tumour progression.	
MAPK3	It is a member of the MAP kinase family. MAP kinases, also known as ERKs, extracellular signal-regulated kinases, physiologically act in a signaling cascade that regulates many cellular processes such as proliferation, differentiation, and cell cycle progression. Diseases especially associated with MAPK3 include B cell lymphoma and bile duct cancer. The evidence for MAPK activation is clearly detectable in thyroid cancer and in melanoma. In fact, MAP kinases are crucial in many cancer types and targeted therapies has been already developed. It is expressed abundantly among healthy thyroid tissue, and it was shown that thyroid hormones induce activation of the MAPK.	GeneCards database [54]<br>Protein Atlas [72]<br>Knauf et al. [73]<br>Lin et al. [74]
BCL7C	BAF chromatin remodeling complex subunit BCL7C. This gene is known to be directly involved in a three-way gene translocation in a Burkitt lymphomas, but the function of this gene has not yet been determined. It is expressed in the thyroid tissue on a medium level.	GeneCards database [54]<br>Protein Atlas [72]
KIF9	Kinesin family member 9 gene product is involved in extracellular matrix disassembly and organelle disassembly. It has been localised in microtubules, podosomes and cellular vesicles. It seems essential for normal male fertility and the motility of sperms. In thyroid tissue it is expressed abundantly, although its function for the thyroid cells remains unclear. In cancer cells high *KIF9* expression was associated with *cancer* progression and in most cases was associated with significantly poor survival, especially among GBM patients.	GeneCards database [54]<br>Protein Atlas [72]<br>Cho et al. [75]
SLC26A6	Solute Carrier Family 26 Member 6 is an anion transporter protein. This particular protein is involved in transporting chloride, oxalate, sulphate and bicarbonate. It is present in the thyroid tissue, but also inner ear and kidneys, among other locations. Diseases associated with SLC26A6 mutations among humans include sialolithiasis and urolithiasis. It has been debated that *SLC26A6* gene might be taken as a novel oncogene in hepatocellular carcinoma, although further research is required to confirm this hypothesis. Moreover, it was reported to be mutated in over 10% of the colorectal *cancer* cell lines.	GeneCards database [54]<br>Cao et al. [76]<br>Alper et al. [77]<br>Zhu et al. [78]
UBA7	Ubiquitin Like Modifier Activating Enzyme 7 forms an important cellular protein complex responsible for targeting abnormal or short-lived proteins for degradation. It is involved in the pathogenesis of leukaemias and lung and breast cancers. One paper	GeneCards database [54]<br>Fontaine et al. [79]<br>Lin et al. [80]<br>Fan et al. [81]

	mentioned it to be up-regulated in follicular thyroid adenoma compared to other thyroid tumours, but no further confirmations can be seen. It is considered to be a candidate *tumour* suppressor gene.	
CACNA2D2	Calcium Voltage-Gated Channel Auxiliary Subunit Alpha2delta 2 – this gene encodes a subunit of the voltage-dependent calcium channel complex. This subunit CACNA2D2 is a receptor for the antiepileptic drug, gabapentin. Mutations in this gene are associated with early infantile epileptic encephalopathy. SNPs in the CACNA2D2 gene are correlated with increased sensitivity to opioid drugs. it was even shown that *CACNA2D2 gene* is overexpressed in breast *cancer* cell lines, whereas several SNPs in this gene have been linked with the NSCLC. No direct evidence in thyroid cancer has been found to date.	GeneCards database [54]<br>Warnier et al. [82]<br>Carboni et al. [83]
TLR9	Toll Like Receptor 9 plays a crucial role in pathogen recognition and activation of innate immunity. It also mediates the production of several cytokines necessary for the development of the immunity. The level of TLR9 protein seems to be increased among autoimmune thyroid diseases patients. It has been hypothesized that innate immune response pathways, including this involving TLR9, might be involved in thyroid carcinogenesis. Surprisingly, most of the analysed thyroid cancer type tissues exhibited similar expression patterns of TLR9. This finding may be proven by the current study too.	GeneCards database [54]<br>Peng et al. [84]<br>Nihon-Yanagi et al. [85]<br>Inoue et al. [86]
BAP1	BRCA1 Associated Protein 1, this gene product is involved in the removal of ubiquitin from other proteins. It plays a role in the regulation of transcription process, but also regulation of cell cycle and cell growth, as well as response to DNA damage and chromatin modifications. Known mutations in this gene are associated with higher risk of tumour development, including increased risk of cancers such as mesothelioma, uveal melanoma or cutaneous melanoma. It has been also associated with Kury-Isidor Syndrome. Moreover, the loss of BAP1 tumour suppression directly contributes to the development of thyroid cancers and possibly also pancreatic tumours.	GeneCards database [54]<br>McDonnell et al. [87]<br>Farid et al. [88]<br>Haugh et al. [89]<br>Gallanis et al. [90]
TYRO3	TYRO3 Protein Tyrosine Kinase; this gene protein product in involved in control of cell survival and proliferation, immunoregulation, phagocytosis and spermatogenesis. Moreover, it has been identified as a potential cell entry factor for important viruses: Ebola and Marburg. There are several diseases associated with the mutations in this gene, including lymphocytic choriomeningitis and one subtype pf the retinitis pigmentosa. It has been connected with thyroid cancer pathogenesis and it has been observed that this receptor was constitutively expressed in thyroid	GeneCards database [54]<br>Avilla et al. [91]<br>Protein Atlas [72]<br>Hsu et al. [92]

	cancer cell, but not normal thyroid cells. Even though the expression in cancer cells is high, it is not a prognostic factor in thyroid cancer. Considering its broad impact, including cell proliferation promotion, survival, tumorigenesis, migration, invasion and even epithelial-mesenchymal transition, as well as chemoresistance in several human cancers, it is considered currently as a potential target for personalised therapy and several clinical trials are ongoing in this field.	
CAPN3	Calpain 3 is a subunit of a large enzyme complex, a major intracellular calcium-sensitive protease, that binds to titin. Muscular dystrophies have been associated with the mutations in this gene. It is expressed in thyroid tissue, both in healthy and pathological conditions, and there are some isolated mentions of gene-gene interactions associated with papillary *thyroid* cancer.	GeneCards database [54]<br>Ao et al. [93]<br>Protein Atlas [72]
TP53BP1	Tumour Protein P53 Binding Protein 1; It forms one of the best cancer-related complexes known to date. The gene TP53BP1 encodes a protein involved in the DNA double-strand break (dsDNA breaks) repair pathway, utilising non-homologous end joining (NHEJ) pathway. It has multiple roles in the DNA damage response processes, including promoting checkpoint signalling if DNA damage is detected, acting as a scaffold for recruitment of the DNA damage-response proteins to the place of damage. It is also involved in immune system functioning during V(D)J recombination and class switch recombination. Apart from cancer, it is also involved in microcephaly development. Recently it has been proposed as a biomarker to differentiate thyroid follicular tumours, using liquid biopsy technique.	GeneCards database [54]<br>Sato et al. [94]<br>Xia et al. [95]<br>Mussazhanova et al. [96]<br>Otsubo et al. [97]<br>Zambrano et al. [98]<br>Luong et al. [99]
EIF3J	Eukaryotic Translation Initiation Factor 3 Subunit J; protein product of this gene participates in the translation initiation by aiding in the recruitment of necessary components to the 40S ribosomal subunit. Expression in thyroid cancer cells seems to be low and the specific function of this protein in thyroid requires further investigation.	GeneCards database [54]<br>Lee et al. [100]<br>Lee et al. [101]<br>Protein Atlas [72]

Table S2. Significant genes located in the LOHs discovered more frequently among follicular thyroid carcinoma patients.

Gene	Description	References
FOXN4	Forkhead Box N4, protein product of this gene is a transcription factor involved in many biological processes, usually as key regulator in development and metabolism. Diseases associated with some variants of this gene include Lymphedema-Distichiasis Syndrome and Retinitis Pigmentosa. Although its function in cancer process is not well understood, it seems to directly activates p53.	GeneCards database [54]<br>Chi et al. [102]<br>Luo et al. [103]
MYL2	Myosin Light Chain 2; this gene encodes a major sarcomeric protein located in striated muscle. It plays a role especially in embryonic heart muscle structure and function, while phosphorylation of the encoded protein is involved in cardiac myosin function in adults. Several variants of this gene are associated with hypertrophic cardiomyopathy disease. Although the specific function of the gene in thyroid remains unknown, it has been suggested that transthyretin maintains muscle homeostasis through the novel pathway of thyroid hormones during myoblast differentiation, which includes MYL2 protein.	GeneCards database [54]<br>MalaCards [104]<br>Weterman et al. [105]<br>Manivannan et al. [106]<br>Claes et al. [107]<br>Lee et al. [108]
PTPN11	Protein Tyrosine Phosphatase Non-Receptor Type 11; the protein encoded by this gene belongs to the tyrosine phosphatase protein family. They are signaling molecules involved in a variety of cellular processes including cell growth, differentiation, mitotic cycle, but also in oncogenic transformation. Among diseases connected with PTPN11 variants are Noonan Syndrome 1 and Juvenile Myelomonocytic Leukemia. Several tyrosine phosphatases are known to be significant in thyroid carcinoma. The expression is usually increased in thyroid carcinoma.	GeneCards database [54]<br>MalaCards [104]<br>Miao et al. [109]<br>Lee et al. [110]<br>Pannone et al. [111]<br>Hu et al. [112]
UBE3B	Ubiquitin Protein Ligase E3B; ubiquitination is an important cellular mechanism used for targeting short-lived or damaged proteins for degradation. There are several non-cancerous diseases related to the *UBE3B* alterations and malfunctioning, such as Angelman Syndrome and Blepharophimosis. It is highly expressed in thyroid tissue and potentially might be related to the TSH levels, although further studies are required.	GeneCards database [54]<br>MalaCards [104]<br>Basel-Vanagaite [113]<br>Protein Atlas [72]<br>Basel-Vanagaite [114]
OAS	2'-5'-Oligoadenylate Synthetase 1-3; interferon-induced OAS gene family protein products plays a key role in innate cellular antiviral response. They are also known to be implicated in other cellular processes like cell growth and apoptosis. Some immunodeficiencies might relate to the OAS genes abnormalities. IFNα induces up-regulation of gene expression in *thyroid* tissue, especially in anaplastic thyroid carcinoma, although the exact contribution of OAS1-3-proteins to the thyroid carcinogenesis remains to be revealed.	GeneCards database [54]<br>Wickenhagen et al. [115]<br>Yamazaki et al. [116]<br>Stefan et al. [117]<br>Poma et al. [118]<br>Hébrant et al. [119]<br>Zhen et al. [120]<br>Jiang et al. [121]

RAD9B	RAD9 Checkpoint Clamp Component B; The protein function encoded by this gene is predicted to be involved in DNA integrity checkpoint signaling and possibly also DNA repair. It has been observed to play a role in cellular response to ionizing radiation. The expression in normal thyroid tissue remains very low.	GeneCards database [54]<br>Protein Atlas [72]<br>Auslander et al. [122]
RASAL1	RAS Protein Activator Like 1; its protein product belongs to the GTPase-activating proteins. It is involved in stimulation of the GTPase activity of normal RAS p21 but not its oncogenic counterpart. It suppresses RAS function. Plays a role in control of cellular proliferation and differentiation. This protein is strongly expressed in endocrine tissues, including thyroid. It seems to be important on thyroid functioning, hypermethylation and abnormalities were mutually exclusive and collectively found in no *thyroid* tumors. In fact, it has been recently identified as a major tumor suppressor gene in thyroid cancer. RASAL1 gene abnormalities have been identified in all types of thyroid cancer. There is a suggestion that RASAL1 mutations might be responsible for certain familial cancers other than Cowden syndrome.	GeneCards database [54]<br>Xing et al. [123]<br>Chang et al. [124]<br>Liu et al. [125]<br>Wang et al. [126]<br>Hińcza et al. [127]<br>Lyssikatos et al. [128]

Table S3. Significant genes located in the LOHs discovered more frequently among follicular thyroid adenoma compared to follicular thyroid cancers patients.

Gene	Description	References
SRC	SRC Proto-Oncogene, its protein product, a tyrosine kinase, plays a role in the regulation of embryonic development and cell growth. Mutations in this gene are known to be involved in human cancer development, especially colon cancer. It is also involved in thyroid cancer pathogenesis, and it has been shown that SRC inhibitors effectively inhibited the signaling transduction pathway in thyroid cancer cells in vitro and in vivo. Its malfunctioning has been reported in papillary thyroid carcinoma, but not in follicular thyroid adenoma.	GeneCards database [54]<br>MalaCards [104]<br>Henderson et al. [129]<br>Beadnell et al. [130]<br>Chan et al. [131]<br>Lee et al. [132]<br>Liu et al. [133]
KAI1	Also known as CD82, is a membrane glycoprotein. The expression of CD82 has been shown to be downregulated in tumour progression and claimed to be a metastasis suppressor. The loss of CD82 expression is associated with poor survival among prostate cancer patients. *CD82* expression was correlated with pTNM status of *thyroid* tumour cells. Its malfunctions have been reported in papillary thyroid carcinoma, but not in follicular thyroid adenoma.	GeneCards database [54]<br>Chen et al. [134]<br>Kim et al. [135]
OR4N4	Olfactory Receptor Family 4 Subfamily N Member 4; The protein product of this gene is an olfactory receptor are responsible for the recognition and G protein-mediated transduction of odorant signals. Interestingly, the olfactory receptor gene family members are among the largest in the human genome. It is expressed in thyroid and testis, both healthy and cancerous tissue, but has not been proven as a prognostic factor. There were significant changes in *OR4N4* gene expression in human *thyroid* epithelial cell lines reported, especially after exposure to high-dose gamma radiation.	GeneCards database [54]<br>Protein Atlas [72]<br>Qiu et al. [136]<br>Bang et al. [137]<br>Weidinger et al. [138]<br>Abaffy et al. [139]
CREBBP	CREB binding protein; its product plays a critical role in embryonic development, growth control, and homeostasis by coupling chromatin remodeling to transcription factor recognition. Among human diseases connected with CREBBP pathogenic variants is Rubinstein-Taybi Syndrome 1**.**	GeneCards database [54]<br>MalaCards [104]
BCL2L1	BCL2 Like 1 protein product is involved in apoptotic processes and members of this family are known to be deregulated in many human cancers, especially B-cell lymphoma. Together with other complex members, it seems to play a role in protection of *thyroid* carcinoma cells against chemotherapy-induced apoptosis.	GeneCards database [54]<br>Mitsiades et al. [140]<br>Wang et al. [141]<br>He et al. [142]<br>Rakhsh-Khorshid et al. [143]
DNMT3B	DNA Methyltransferase 3 Beta; Protein product is necessary for genome-wide de novo methylation, but also for the establishment of DNA methylation patterns during development. It has been observed malfunctioning in autoimmune thyroid diseases, but	GeneCards database [54]<br>Cai et al. [144]<br>Kyono et al. [145]<br>Coppedè et al. [146]<br>Zafon et al. [147]

	also in several thyroid malignancies. Its direct role in thyroid tumours remain unknown.	Arakawa et al. [148]<br>Cai et al. [149]<br>Wojcicka et al. [150]
MMP24	Matrix Metallopeptidase 24; this gene encodes an enzyme involved in the breakdown of extracellular matrix, ECM, under normal physiological processes, especially during embryonic development and tissue remodeling. It is also important in disease processes, such as arthritis and cancer metastasis, including thyroid tumours. Interestingly, its variants have been associated with asymptomatic Dengue disease.	GeneCards database [54]<br>MalaCards [64]<br>Lee et al. [151]<br>Gobin et al. [152]<br>Rodrigues et al. [153]<br>Bialek et al. [154]

51. Odermatt, A.; Barton, K.; Khanna, V.K.; Mathieu, J.; Escolar, D.; Kuntzer, T.; Karpati, G.; MacLennan, D.H. The Mutation of Pro789 to Leu Reduces the Activity of the Fast-Twitch Skeletal Muscle Sarco(Endo)Plasmic Reticulum Ca2+ ATPase (SERCA1) and Is Associated with Brody Disease. *Hum Genet* **2000**, *106*, 482–491, doi:10.1007/s004390000297.
52. Expression of ATP2A1 in Cancer - Summary - The Human Protein Atlas Available online: https://www.proteinatlas.org/ENSG00000196296-ATP2A1/pathology (accessed on 31 July 2022).
53. Bloise, F.F.; Cordeiro, A.; Ortiga-Carvalho, T.M. Role of Thyroid Hormone in Skeletal Muscle Physiology. *J Endocrinol* **2018**, *236*, R57–R68, doi:10.1530/JOE-16-0611.
54. GeneCards Database.
55. Hartong, R.; Wang, N.; Kurokawa, R.; Lazar, M.A.; Glass, C.K.; Apriletti, J.W.; Dillmann, W.H. Delineation of Three Different Thyroid Hormone-Response Elements in Promoter of Rat Sarcoplasmic Reticulum Ca2+ATPase Gene. Demonstration That Retinoid X Receptor Binds 5′ to Thyroid Hormone Receptor in Response Element 1. *Journal of Biological Chemistry* **1994**, *269*, 13021–13029, doi:10.1016/S0021-9258(18)99978-3.
56. Muller, A.; Vanderlinden, G.C.; Zuidwijk, M.J.; Simonides, W.S.; Vanderlaarse, W.J.; Vanhardeveld, C. Differential Effects of Thyroid Hormone on the Expression of Sarcoplasmic Reticulum Ca2+-ATPase Isoforms in Rat Skeletal Muscle Fibers. *Biochemical and Biophysical Research Communications* **1994**, *203*, 1035–1042, doi:10.1006/bbrc.1994.2286.
57. Simonides, W.S.; Brent, G.A.; Thelen, M.M.; Linden, C.G. van der; Larsen, P.R.; Hardeveld, C. van Characterization of the Promoter of the Rat Sarcoplasmic Endoplasmic Reticulum Ca2+-ATPase 1 Gene and Analysis of Thyroid Hormone Responsiveness *. *Journal of Biological Chemistry* **1996**, *271*, 32048–32056, doi:10.1074/jbc.271.50.32048.
58. Dang, D.; Rao, R. Calcium-ATPases: Gene Disorders and Dysregulation in Cancer. *Biochimica et Biophysica Acta (BBA) - Molecular Cell Research* **2016**, *1863*, 1344–1350, doi:10.1016/j.bbamcr.2015.11.016.
59. He, W.; Wang, B.; Mu, K.; Zhang, J.; Yang, Y.; Yao, W.; Li, S.; Zhang, J. Association of Single-Nucleotide Polymorphisms in the IL27 Gene with Autoimmune Thyroid Diseases. *Endocrine Connections* **2019**, *8*, 173–181, doi:10.1530/EC-18-0370.
60. Saeed, M.-H.; Kurosh, K.; Zahra, A.; Hossein, D.M.; Davood, R.; Ataollahi, M.R. Decreased Serum Levels of IL-27and IL-35 in Patients with Graves Disease. *Archives of Endocrinology and Metabolism* **2020**, doi:10.20945/2359-3997000000227.
61. Nie, X.; Yuan, F.; Chen, P.; Pu, Y.; Zhu, J.; Wang, Y.; Xiao, X.; Che, G.; Gao, L.; Zhang, L. Association between *IL-27* Gene Polymorphisms and Risk of Papillary Thyroid Carcinoma. *Biomarkers in Medicine* **2017**, *11*, 141–149, doi:10.2217/bmm-2016-0283.
62. Xi, C.; Zhang, G.-Q.; Sun, Z.-K.; Song, H.-J.; Shen, C.-T.; Chen, X.-Y.; Sun, J.-W.; Qiu, Z.-L.; Luo, Q.-Y. Interleukins in Thyroid Cancer: From Basic Researches to Applications in Clinical Practice. *Front. Immunol.* **2020**, *11*, 1124, doi:10.3389/fimmu.2020.01124.

63. Jia, H.; Dilger, P.; Bird, C.; Wadhwa, M. IL-27 Promotes Proliferation of Human Leukemic Cell Lines Through the MAPK/ERK Signaling Pathway and Suppresses Sensitivity to Chemotherapeutic Drugs. *Journal of Interferon & Cytokine Research* **2016**, *36*, 302–316, doi:10.1089/jir.2015.0091.
64. Larousserie, F.; Bardel, E.; Coulomb L'Herminé, A.; Canioni, D.; Brousse, N.; Kastelein, R.; Devergne, O. Variable Expression of Epstein–Barr Virus-Induced Gene 3 during Normal B-Cell Differentiation and among B-Cell Lymphomas. *J. Pathol.* **2006**, *209*, 360–368, doi:10.1002/path.1995.
65. Gonin, J.; Carlotti, A.; Dietrich, C.; Audebourg, A.; Radenen-Bussière, B.; Caignard, A.; Avril, M.-F.; Vacher-Lavenu, M.-C.; Larousserie, F.; Devergne, O. Expression of IL-27 by Tumor Cells in InvasCutaneous and Metastatic Melanomas. *PLoS ONE* **2013**, *8*, e75694, doi:10.1371/journal.pone.0075694.
66. Kourko, O.; Seaver, K.; Odoardi, N.; Basta, S.; Gee, K. IL-27, IL-30, and IL-35: A Cytokine Triumvirate in Cancer. *Front. Oncol.* **2019**, *9*, 969, doi:10.3389/fonc.2019.00969.
67. Pisarev, M.A.; Thomasz, L.; Juvenal, G.J. Role of Transforming Growth Factor Beta in the Regulation of Thyroid Function and Growth. *Thyroid* **2009**, *19*, 881–892, doi:10.1089/thy.2007.0303.
68. Kardalas, E.; Sakkas, E.; Ruchala, M.; Macut, D.; Mastorakos, G. The Role of Transforming Growth Factor Beta in Thyroid Autoimmunity: Current Knowledge and Future Perspectives. *Rev Endocr Metab Disord* **2022**, *23*, 431–447, doi:10.1007/s11154-021-09685-7.
69. Mincione, G.; Di Marcantonio, M.C.; Tarantelli, C.; D'Inzeo, S.; Nicolussi, A.; Nardi, F.; Donini, C.F.; Coppa, A. EGF and TGF- <b/> 1 Effects on Thyroid Function. *Journal of Thyroid Research* **2011**, *2011*, 1–13, doi:10.4061/2011/431718.
70. Grubeck-Loebenstein, B.; Buchan, G.; Sadeghi, R.; Kissonerghis, M.; Londei, M.; Turner, M.; Pirich, K.; Roka, R.; Niederle, B.; Kassal, H. Transforming Growth Factor Beta Regulates Thyroid Growth. Role in the Pathogenesis of Nontoxic Goiter. *J. Clin. Invest.* **1989**, *83*, 764–770, doi:10.1172/JCI113955.
71. Wikipathways MAPK Pathway in Congenital Thyroid Cancer (Homo Sapiens).
72. Protein Atlas.
73. Knauf, J.A.; Fagin, J.A. Role of MAPK Pathway Oncoproteins in Thyroid Cancer Pathogenesis and as Drug Targets. *Current Opinion in Cell Biology* **2009**, *21*, 296–303, doi:10.1016/j.ceb.2009.01.013.
74. Lin, H.-Y.; Davis, F.B.; Gordinier, J.K.; Martino, L.J.; Davis, P.J. Thyroid Hormone Induces Activation of Mitogen-Activated Protein Kinase in Cultured Cells. *American Journal of Physiology-Cell Physiology* **1999**, *276*, C1014–C1024, doi:10.1152/ajpcell.1999.276.5.C1014.
75. Cho, S.Y.; Kim, S.; Kim, G.; Singh, P.; Kim, D.W. Integrative Analysis of KIF4A, 9, 18A, and 23 and Their Clinical Significance in Low-Grade Glioma and Glioblastoma. *Sci Rep* **2019**, *9*, 4599, doi:10.1038/s41598-018-37622-3.
76. Cao, J.; Wang, P.; Chen, J.; He, X. Systemic Characterization of the SLC Family Genes Reveals SLC26A6 as a Novel Oncogene in Hepatocellular Carcinoma. *Transl Cancer Res* **2021**, *10*, 2882–2894, doi:10.21037/tcr-20-1751.
77. Alper, S.L.; Sharma, A.K. The SLC26 Gene Family of Anion Transporters and Channels. *Molecular Aspects of Medicine* **2013**, *34*, 494–515, doi:10.1016/j.mam.2012.07.009.
78. Zhu, Y.; Huang, Y.; Chen, L.; Guo, L.; Wang, L.; Li, M.; Liang, Y. Up-Regulation of SLC26A6 in Hepatocellular Carcinoma and Its Diagnostic and Prognostic Significance. *Crit Rev Eukaryot Gene Expr* **2021**, *31*, 79–94, doi:10.1615/CritRevEukaryotGeneExpr.2021039703.
79. Fontaine, J.-F.; Mirebeau-Prunier, D.; Franc, B.; Triau, S.; Rodien, P.; Houlgatte, R.; Malthièry, Y.; Savagner, F. Microarray Analysis Refines Classification of Non-Medullary Thyroid Tumours of Uncertain Malignancy. *Oncogene* **2008**, *27*, 2228–2236, doi:10.1038/sj.onc.1210853.
80. Lin, M.; Li, Y.; Qin, S.; Jiao, Y.; Hua, F. Ubiquitin-like Modifier-activating Enzyme 7 as a Marker for the Diagnosis and Prognosis of Breast Cancer. *Oncol Lett* **2020**, doi:10.3892/ol.2020.11406.

81. Fan, J.-B.; Miyauchi, S.; Xu, H.-Z.; Liu, D.; Kim, L.J.Y.; Burkart, C.; Cheng, H.; Arimoto, K.; Yan, M.; Zhou, Y.; et al. Type I Interferon Regulates a Coordinated Gene Network to Enhance Cytotoxic T Cell–Mediated Tumor Killing. *Cancer Discovery* **2020**, *10*, 382–393, doi:10.1158/2159-8290.CD-19-0608.
82. Warnier, M.; Roudbaraki, M.; Derouiche, S.; Delcourt, P.; Bokhobza, A.; Prevarskaya, N.; Mariot, P. CACNA2D2 Promotes Tumorigenesis by Stimulating Cell Proliferation and Angiogenesis. *Oncogene* **2015**, *34*, 5383–5394, doi:10.1038/onc.2014.467.
83. Carboni, G.L.; Gao, B.; Nishizaki, M.; Xu, K.; Minna, J.D.; Roth, J.A.; Ji, L. CACNA2D2-Mediated Apoptosis in NSCLC Cells Is Associated with Alterations of the Intracellular Calcium Signaling and Disruption of Mitochondria Membrane Integrity. *Oncogene* **2003**, *22*, 615–626, doi:10.1038/sj.onc.1206134.
84. Peng, S.; Li, C.; Wang, X.; Liu, X.; Han, C.; Jin, T.; Liu, S.; Zhang, X.; Zhang, H.; He, X.; et al. Increased Toll-Like Receptors Activity and TLR Ligands in Patients with Autoimmune Thyroid Diseases. *Front. Immunol.* **2016**, *7*, 578, doi:10.3389/fimmu.2016.00578.
85. Nihon-Yanagi, Y.; Wakayama, M.; Tochigi, N.; Saito, F.; Ogata, H.; Shibuya, K. Immunohistochemical Analysis of Toll-Like Receptors, MyD88, and TRIF in Human Papillary Thyroid Carcinoma and Anaplastic Thyroid Carcinoma. *Journal of Thyroid Research* **2021**, *2021*, 1–12, doi:10.1155/2021/4226491.
86. Inoue, N.; Katsumata, Y.; Watanabe, M.; Ishido, N.; Manabe, Y.; Watanabe, A.; Masutani, R.; Hidaka, Y.; Iwatani, Y. Polymorphisms and Expression of Toll-like Receptors in Autoimmune Thyroid Diseases. *Autoimmunity* **2017**, *50*, 182–191, doi:10.1080/08916934.2016.1261835.
87. McDonnell, K.J.; Gallanis, G.T.; Heller, K.A.; Melas, M.; Idos, G.E.; Culver, J.O.; Martin, S.-E.; Peng, D.H.; Gruber, S.B. A Novel BAP1 Mutation Is Associated with Melanocytic Neoplasms and Thyroid Cancer. *Cancer Genetics* **2016**, *209*, 75–81, doi:10.1016/j.cancergen.2015.12.007.
88. Farid RM, Abd El Atti RM, Abd Raboh NM Immunohistochemical Expression of the Cancer Predisposition Gene BRCA1-Associated Protein 1 in Thyroid and Lung Carcinoma. *Egypt J Pathol*.
89. Haugh, A.M.; Njauw, C.-N.; Bubley, J.A.; Verzì, A.E.; Zhang, B.; Kudalkar, E.; VandenBoom, T.; Walton, K.; Swick, B.L.; Kumar, R.; et al. Genotypic and Phenotypic Features of BAP1 Cancer Syndrome: A Report of 8 New Families and Review of Cases in the Literature. *JAMA Dermatol* **2017**, *153*, 999, doi:10.1001/jamadermatol.2017.2330.
90. Gallanis, G.T.; Heller, K.A.; Melas, E.-M.; Gruber, S.B. Abstract 3522: A Novel BAP1 Mutation Is Associated with Melanocytic Neoplasms and Thyroid and Pancreatic Cancers. *Cancer Research* **2014**, *74*, 3522–3522, doi:10.1158/1538-7445.AM2014-3522.
91. Avilla, E.; Guarino, V.; Visciano, C.; Liotti, F.; Svelto, M.; Krishnamoorthy, G.; Franco, R.; Melillo, R.M. Activation of TYRO3/AXL Tyrosine Kinase Receptors in Thyroid Cancer. *Cancer Research* **2011**, *71*, 1792–1804, doi:10.1158/0008-5472.CAN-10-2186.
92. Hsu, P.-L.; Jou, J.; Tsai, S.-J. TYRO3: A Potential Therapeutic Target in Cancer. *Exp Biol Med (Maywood)* **2019**, *244*, 83–99, doi:10.1177/1535370219828195.
93. Ao, Z.; Chen, Y.; Lu, J.; Shen, J.; Peng, L.; Lin, X.; Peng, C.; Zeng, C.; Wang, X.; Zhou, R.; et al. Identification of Potential Functional Genes in Papillary Thyroid Cancer by Co-expression Network Analysis. *Oncol Lett* **2018**, doi:10.3892/ol.2018.9306.
94. Sato, A.; Matsuda, K.; Motoyama, T.; Mussazhanova, Z.; Otsubo, R.; Kondo, H.; Akazawa, Y.; Higuchi, M.; Suzuki, A.; Hirokawa, M.; et al. 53BP1 Expression as a Biomarker to Differentiate Thyroid Follicular Tumors. *Endocrine Connections* **2021**, *10*, 309–315, doi:10.1530/EC-20-0630.
95. Xia, Z.; Morales, J.C.; Dunphy, W.G.; Carpenter, P.B. Negative Cell Cycle Regulation and DNA Damage-Inducible Phosphorylation of the BRCT Protein 53BP1. *Journal of Biological Chemistry* **2001**, *276*, 2708–2718, doi:10.1074/jbc.M007665200.
96. Mussazhanova, Z.; Matsuda, K.; Naruke, Y.; Mitsutake, N.; Stanojevic, B.; Rougounovitch, T.; Saenko, V.; Suzuki, K.; Nishihara, E.; Hirokawa, M.; et al. Significance of P53-Binding Protein 1

(53BP1) Expression in Thyroid Papillary Microcarcinoma: Association with *BRAF* ^{V600E} Mutation Status. *Histopathology* **2013**, n/a-n/a, doi:10.1111/his.12233.
97. Otsubo, R.; Matsuda, K.; Mussazhanova, Z.; Sato, A.; Matsumoto, M.; Yano, H.; Oikawa, M.; Kondo, H.; Ito, M.; Miyauchi, A.; et al. A Novel Diagnostic Method for Thyroid Follicular Tumors Based on Immunofluorescence Analysis of P53-Binding Protein 1 Expression: Detection of Genomic Instability. *Thyroid* **2019**, *29*, 657–665, doi:10.1089/thy.2018.0548.
98. Zambrano, A.; García-Carpizo, V.; Gallardo, M.E.; Villamuera, R.; Gómez-Ferrería, M.A.; Pascual, A.; Buisine, N.; Sachs, L.M.; Garesse, R.; Aranda, A. The Thyroid Hormone Receptor β Induces DNA Damage and Premature Senescence. *Journal of Cell Biology* **2014**, *204*, 129–146, doi:10.1083/jcb.201305084.
99. Luong, T.M.H.; Matsuda, K.; Niino, D.; Kurohama, H.; Ito, M.; Nakashima, M. Significance of Abnormal 53BP1 Expression as a Novel Molecular Pathologic Parameter of Follicular-Shaped B-Cell Lymphoid Lesions in Human Digestive Tract. *Sci Rep* **2021**, *11*, 3074, doi:10.1038/s41598-021-82867-0.
100. Lee, A.S.Y.; Kranzusch, P.J.; Cate, J.H.D. EIF3 Targets Cell-Proliferation Messenger RNAs for Translational Activation or Repression. *Nature* **2015**, *522*, 111–114, doi:10.1038/nature14267.
101. Lee, A.S.Y.; Kranzusch, P.J.; Doudna, J.A.; Cate, J.H.D. EIF3d Is an MRNA Cap-Binding Protein That Is Required for Specialized Translation Initiation. *Nature* **2016**, *536*, 96–99, doi:10.1038/nature18954.
102. Chi, N.C.; Shaw, R.M.; De Val, S.; Kang, G.; Jan, L.Y.; Black, B.L.; Stainier, D.Y.R. Foxn4 Directly Regulates *Tbx2b* Expression and Atrioventricular Canal Formation. *Genes Dev.* **2008**, *22*, 734–739, doi:10.1101/gad.1629408.
103. Luo, H.; Jin, K.; Xie, Z.; Qiu, F.; Li, S.; Zou, M.; Cai, L.; Hozumi, K.; Shima, D.T.; Xiang, M. Forkhead Box N4 (Foxn4) Activates Dll4-Notch Signaling to Suppress Photoreceptor Cell Fates of Early Retinal Progenitors. *Proc. Natl. Acad. Sci. U.S.A.* **2012**, *109*, doi:10.1073/pnas.1115767109.
104. MalaCards.
105. Weterman, M.A.J.; Barth, P.G.; van Spaendonck-Zwarts, K.Y.; Aronica, E.; Poll-The, B.-T.; Brouwer, O.F.; van Tintelen, J.P.; Qahar, Z.; Bradley, E.J.; de Wissel, M.; et al. Recessive MYL2 Mutations Cause Infantile Type I Muscle Fibre Disease and Cardiomyopathy. *Brain* **2013**, *136*, 282–293, doi:10.1093/brain/aws293.
106. Manivannan, S.N.; Darouich, S.; Masmoudi, A.; Gordon, D.; Zender, G.; Han, Z.; Fitzgerald-Butt, S.; White, P.; McBride, K.L.; Kharrat, M.; et al. Novel Frameshift Variant in MYL2 Reveals Molecular Differences between Dominant and Recessive Forms of Hypertrophic Cardiomyopathy. *PLoS Genet* **2020**, *16*, e1008639, doi:10.1371/journal.pgen.1008639.
107. Claes, G.R.F.; van Tienen, F.H.J.; Lindsey, P.; Krapels, I.P.C.; Helderman-van den Enden, A.T.J.M.; Hoos, M.B.; Barrois, Y.E.G.; Janssen, J.W.H.; Paulussen, A.D.C.; Sels, J.-W.E.M.; et al. Hypertrophic Remodelling in Cardiac Regulatory Myosin Light Chain (*MYL2*) Founder Mutation Carriers. *Eur Heart J* **2016**, *37*, 1815–1822, doi:10.1093/eurheartj/ehv522.
108. Lee, E.J.; Shaikh, S.; Choi, D.; Ahmad, K.; Baig, M.H.; Lim, J.H.; Lee, Y.-H.; Park, S.J.; Kim, Y.-W.; Park, S.-Y.; et al. Transthyretin Maintains Muscle Homeostasis through the Novel Shuttle Pathway of Thyroid Hormones during Myoblast Differentiation. *Cells* **2019**, *8*, 1565, doi:10.3390/cells8121565.
109. Miao, H.; Burnett, E.; Kinch, M.; Simon, E.; Wang, B. Activation of EphA2 Kinase Suppresses Integrin Function and Causes Focal-Adhesion-Kinase Dephosphorylation. *Nat Cell Biol* **2000**, *2*, 62–69, doi:10.1038/35000008.
110. Lee, H.-H.; Chang, Z.-F. Regulation of RhoA-Dependent ROCKII Activation by Shp2. *Journal of Cell Biology* **2008**, *181*, 999–1012, doi:10.1083/jcb.200710187.
111. Pannone, L.; Bocchinfuso, G.; Flex, E.; Rossi, C.; Baldassarre, G.; Lissewski, C.; Pantaleoni, F.; Consoli, F.; Lepri, F.; Magliozzi, M.; et al. Structural, Functional, and Clinical Characterization of a Novel *PTPN11* Mutation Cluster Underlying Noonan Syndrome: HUMAN MUTATION. *Human Mutation* **2017**, *38*, 451–459, doi:10.1002/humu.23175.

112. Hu, Z.-Q.; Ma, R.; Zhang, C.-M.; Li, J.; Li, L.; Hu, Z.-T.; Gao, Q.; Li, W.-M. Expression and Clinical Significance of Tyrosine Phosphatase SHP2 in Thyroid Carcinoma. *Oncology Letters* **2015**, *10*, 1507–1512, doi:10.3892/ol.2015.3479.
113. Basel-Vanagaite, L.; Dallapiccola, B.; Ramirez-Solis, R.; Segref, A.; Thiele, H.; Edwards, A.; Arends, M.J.; Miró, X.; White, J.K.; Désir, J.; et al. Deficiency for the Ubiquitin Ligase UBE3B in a Blepharophimosis-Ptosis-Intellectual-Disability Syndrome. *The American Journal of Human Genetics* **2012**, *91*, 998–1010, doi:10.1016/j.ajhg.2012.10.011.
114. Basel-Vanagaite, L.; Yilmaz, R.; Tang, S.; Reuter, M.S.; Rahner, N.; Grange, D.K.; Mortenson, M.; Koty, P.; Feenstra, H.; Farwell Gonzalez, K.D.; et al. Expanding the Clinical and Mutational Spectrum of Kaufman Oculocerebrofacial Syndrome with Biallelic UBE3B Mutations. *Hum Genet* **2014**, *133*, 939–949, doi:10.1007/s00439-014-1436-2.
115. Wickenhagen, A.; Sugrue, E.; Lytras, S.; Kuchi, S.; Noerenberg, M.; Turnbull, M.L.; Loney, C.; Herder, V.; Allan, J.; Jarmson, I.; et al. A Prenylated DsRNA Sensor Protects against Severe COVID-19. *Science* **2021**, *374*, eabj3624, doi:10.1126/science.abj3624.
116. Yamazaki, K.; Suzuki, K.; Yamada, E.; Yamada, T.; Takeshita, F.; Matsumoto, M.; Mitsuhashi, T.; Obara, T.; Takano, K.; Sato, K. Suppression of Iodide Uptake and Thyroid Hormone Synthesis with Stimulation of the Type I Interferon System by Double-Stranded Ribonucleic Acid in Cultured Human Thyroid Follicles. *Endocrinology* **2007**, *148*, 3226–3235, doi:10.1210/en.2006-1638.
117. Stefan, M.; Wei, C.; Lombardi, A.; Li, C.W.; Concepcion, E.S.; Inabnet, W.B.; Owen, R.; Zhang, W.; Tomer, Y. Genetic–Epigenetic Dysregulation of Thymic TSH Receptor Gene Expression Triggers Thyroid Autoimmunity. *Proc. Natl. Acad. Sci. U.S.A.* **2014**, *111*, 12562–12567, doi:10.1073/pnas.1408821111.
118. Poma, A.M.; Basolo, A.; Bonuccelli, D.; Proietti, A.; Macerola, E.; Ugolini, C.; Torregrossa, L.; Alì, G.; Giannini, R.; Vignali, P.; et al. Activation of Type I and Type II Interferon Signaling in SARS-CoV-2-Positive Thyroid Tissue of Patients Dying from COVID-19. *Thyroid* **2021**, *31*, 1766–1775, doi:10.1089/thy.2021.0345.
119. Hébrant, A.; Dom, G.; Dewaele, M.; Andry, G.; Trésallet, C.; Leteurtre, E.; Dumont, J.E.; Maenhaut, C. MRNA Expression in Papillary and Anaplastic Thyroid Carcinoma: Molecular Anatomy of a Killing Switch. *PLoS ONE* **2012**, *7*, e37807, doi:10.1371/journal.pone.0037807.
120. Zhen, J.; Song, Z.; Su, W.; Zeng, Q.-C.; Li, J.; Sun, Q. Integrated Analysis of RNA-Binding Proteins in Thyroid Cancer. *PLoS ONE* **2021**, *16*, e0247836, doi:10.1371/journal.pone.0247836.
121. Jiang, Y.; Zhang, P.; Li, L.-P.; He, Y.-C.; Gao, R.; Gao, Y.-F. Identification of Novel Thyroid Cancer-Related Genes and Chemicals Using Shortest Path Algorithm. *BioMed Research International* **2015**, *2015*, 1–8, doi:10.1155/2015/964795.
122. Auslander, N.; Wolf, Y.I.; Koonin, E.V. Interplay between DNA Damage Repair and Apoptosis Shapes Cancer Evolution through Aneuploidy and Microsatellite Instability. *Nat Commun* **2020**, *11*, 1234, doi:10.1038/s41467-020-15094-2.
123. Xing, M. RASAL1 in Thyroid Cancer: Promise From a New Friend. *The Journal of Clinical Endocrinology & Metabolism* **2014**, *99*, 3619–3621, doi:10.1210/jc.2014-2645.
124. Chang, R.-X.; Cui, A.-L.; Dong, L.; Guan, S.-P.; Jiang, L.-Y.; Miao, C.-X. Overexpression of RASAL1 Indicates Poor Prognosis and Promotes Invasion of Ovarian Cancer. *Open Life Sciences* **2019**, *14*, 133–140, doi:10.1515/biol-2019-0015.
125. Liu, D.; Yang, C.; Bojdani, E.; Murugan, A.K.; Xing, M. Identification of RASAL1 as a Major Tumor Suppressor Gene in Thyroid Cancer. *JNCI Journal of the National Cancer Institute* **2013**, *105*, 1617–1627, doi:10.1093/jnci/djt249.
126. Wang, G.; Li, Z.; Li, X.; Zhang, C.; Peng, L. RASAL1 Induces to Downregulate the SCD1, Leading to Suppression of Cell Proliferation in Colon Cancer via LXRα/SREBP1c Pathway. *Biol Res* **2019**, *52*, 60, doi:10.1186/s40659-019-0268-x.
127. Hińcza, K.; Kowalik, A.; Kowalska, A. Current Knowledge of Germline Genetic Risk Factors for the Development of Non-Medullary Thyroid Cancer. *Genes* **2019**, *10*, 482, doi:10.3390/genes10070482.

128. Lyssikatos, C.; Quezado, M.M.; Faucz, F.R.; Angelousi, A.; Nasiri-Ansari, N.; Stratakis, C.A.; Kassi, E. A Rare Case of Medullary Thyroid Cancer, Mesothelioma and Meningioma, Due to APC and RASAL1 Mutations. *EJEA* **2017**, doi:10.1530/endoabs.49.GP236.
129. Henderson, Y.C.; Toro-Serra, R.; Chen, Y.; Ryu, J.; Frederick, M.J.; Zhou, G.; Gallick, G.E.; Lai, S.Y.; Clayman, G.L. Src Inhibitors in Suppression of Papillary Thyroid Carcinoma Growth: Effects of SRC Inhibitors in PTC. *Head Neck* **2014**, *36*, 375–384, doi:10.1002/hed.23316.
130. Beadnell, T.C.; Nassar, K.W.; Rose, M.M.; Clark, E.G.; Danysh, B.P.; Hofmann, M.-C.; Pozdeyev, N.; Schweppe, R.E. Src-Mediated Regulation of the PI3K Pathway in Advanced Papillary and Anaplastic Thyroid Cancer. *Oncogenesis* **2018**, *7*, 23, doi:10.1038/s41389-017-0015-5.
131. Chan, C.M.; Jing, X.; Pike, L.A.; Zhou, Q.; Lim, D.-J.; Sams, S.B.; Lund, G.S.; Sharma, V.; Haugen, B.R.; Schweppe, R.E. Targeted Inhibition of Src Kinase with Dasatinib Blocks Thyroid Cancer Growth and Metastasis. *Clinical Cancer Research* **2012**, *18*, 3580–3591, doi:10.1158/1078-0432.CCR-11-3359.
132. Lee, W.K.; Kim, W.G.; Fozzatti, L.; Park, S.; Zhao, L.; Willingham, M.C.; Lonard, D.; O'Malley, B.W.; Cheng, S. Steroid Receptor Coactivator-3 as a Target for Anaplastic Thyroid Cancer. *Endocrine-Related Cancer* **2020**, *27*, 209–220, doi:10.1530/ERC-19-0482.
133. Liu, Z.; Falola, J.; Zhu, X.; Gu, Y.; Kim, L.T.; Sarosi, G.A.; Anthony, T.; Nwariaku, F.E. Antiproliferative Effects of Src Inhibition on Medullary Thyroid Cancer. *The Journal of Clinical Endocrinology & Metabolism* **2004**, *89*, 3503–3509, doi:10.1210/jc.2003-031917.
134. Chen, Z. CD82, but Not CD63, Is Linked to Cellular Invasiveness in Human Thyroid Carcinoma. **2007**, Online-Ressource, Text + Image (kB), doi:10.25673/2636.
135. Kim, T.; Kim, Y.; Kwon, H.J. Expression of CD9 and CD82 in Papillary Thyroid Microcarcinoma and Its Prognostic Significance. *Endokrynologia Polska* **2019**, *70*, 224–231, doi:10.5603/EP.a2019.0009.
136. Qiu, K.; Li, K.; Zeng, T.; Liao, Y.; Min, J.; Zhang, N.; Peng, M.; Kong, W.; Chen, L. Integrative Analyses of Genes Associated with Hashimoto's Thyroiditis. *Journal of Immunology Research* **2021**, *2021*, 1–9, doi:10.1155/2021/8263829.
137. Bang, H.S.; Choi, M.H.; Kim, C.S.; Choi, S.J. Gene Expression Profiling in Undifferentiated Thyroid Carcinoma Induced by High-Dose Radiation. *Journal of Radiation Research* **2016**, *57*, 238–249, doi:10.1093/jrr/rrw002.
138. Weidinger, D.; Jovancevic, N.; Zwanziger, D.; Theurer, S.; Hönes, J.; Führer, D.; Hatt, H. Functional Characterization of Olfactory Receptors in the Thyroid Gland. *Front. Physiol.* **2021**, *12*, 676907, doi:10.3389/fphys.2021.676907.
139. Abaffy, T. Human Olfactory Receptors Expression and Their Role in Non-Olfactory Tissues Â A Mini-Review. *J Pharmacogenomics Pharmacoproteomics* **2015**, *06*, doi:10.4172/2153-0645.1000152.
140. Mitsiades, C.S.; Hayden, P.; Kotoula, V.; McMillin, D.W.; McMullan, C.; Negri, J.; Delmore, J.E.; Poulaki, V.; Mitsiades, N. Bcl-2 Overexpression in Thyroid Carcinoma Cells Increases Sensitivity to Bcl-2 Homology 3 Domain Inhibition. *The Journal of Clinical Endocrinology & Metabolism* **2007**, *92*, 4845–4852, doi:10.1210/jc.2007-0942.
141. Wang, Q.; Shen, Y.; Ye, B.; Hu, H.; Fan, C.; Wang, T.; Zheng, Y.; Lv, J.; Ma, Y.; Xiang, M. Gene Expression Differences between Thyroid Carcinoma, Thyroid Adenoma and Normal Thyroid Tissue. *Oncol Rep* **2018**, doi:10.3892/or.2018.6717.
142. He, W.; Qi, B.; Zhou, Q.; Lu, C.; Huang, Q.; Xian, L.; Chen, M. Key Genes and Pathways in Thyroid Cancer Based on Gene Set Enrichment Analysis. *Oncology Reports* **2013**, *30*, 1391–1397, doi:10.3892/or.2013.2557.
143. Rakhsh-Khorshid, H.; Samimi, H.; Torabi, S.; Sajjadi-Jazi, S.M.; Samadi, H.; Ghafouri, F.; Asgari, Y.; Haghpanah, V. Network Analysis Reveals Essential Proteins That Regulate Sodium-Iodide Symporter Expression in Anaplastic Thyroid Carcinoma. *Sci Rep* **2020**, *10*, 21440, doi:10.1038/s41598-020-78574-x.

144. Cai, T.; Zhang, J.; Wang, X.; Song, R.; Qin, Q.; Muhali, F.; Zhou, J.; Xu, J.; Zhang, J. Gene-Gene and Gene-Sex Epistatic Interactions of *DNMT1*, *DNMT3A* and *DNMT3B* in Autoimmune Thyroid Disease. *Endocr J* **2016**, *63*, 643–653, doi:10.1507/endocrj.EJ15-0596.
145. Kyono, Y.; Sachs, L.M.; Bilesimo, P.; Wen, L.; Denver, R.J. Developmental and Thyroid Hormone Regulation of the DNA Methyltransferase 3a Gene in Xenopus Tadpoles. *Endocrinology* **2016**, *157*, 4961–4972, doi:10.1210/en.2016-1465.
146. Coppedè, F. Epigenetics and Autoimmune Thyroid Diseases. *Front. Endocrinol.* **2017**, *8*, 149, doi:10.3389/fendo.2017.00149.
147. Zafon, C.; Gil, J.; Pérez-González, B.; Jordà, M. DNA Methylation in Thyroid Cancer. *Endocrine-Related Cancer* **2019**, *26*, R415–R439, doi:10.1530/ERC-19-0093.
148. Arakawa, Y.; Watanabe, M.; Inoue, N.; Sarumaru, M.; Hidaka, Y.; Iwatani, Y. Association of Polymorphisms in *DNMT1, DNMT3A, DNMT3B, MTHFR* and *MTRR* Genes with Global DNA Methylation Levels and Prognosis of Autoimmune Thyroid Disease. *Clinical and Experimental Immunology* **2012**, *170*, 194–201, doi:10.1111/j.1365-2249.2012.04646.x.
149. Cai, T.; Muhali, F.; Song, R.; Qin, Q.; Wang, X.; Shi, L.; Jiang, W.; Xiao, L.; Li, D.; Zhang, J. Genome-Wide DNA Methylation Analysis in Graves' Disease. *Genomics* **2015**, *105*, 204–210, doi:10.1016/j.ygeno.2015.01.001.
150. Wojcicka, A.; Piekielko–Witkowska, A.; Kedzierska, H.; Rybicka, B.; Poplawski, P.; Boguslawska, J.; Master, A.; Nauman, A. Epigenetic Regulation of Thyroid Hormone Receptor Beta in Renal Cancer. *PLoS ONE* **2014**, *9*, e97624, doi:10.1371/journal.pone.0097624.
151. Lee, J.; Hwang, J.-A.; Lee, E.K. Recent Progress of Genome Study for Anaplastic Thyroid Cancer. *Genomics Inform* **2013**, *11*, 68, doi:10.5808/GI.2013.11.2.68.
152. Gobin, E.; Bagwell, K.; Wagner, J.; Mysona, D.; Sandirasegarane, S.; Smith, N.; Bai, S.; Sharma, A.; Schleifer, R.; She, J.-X. A Pan-Cancer Perspective of Matrix Metalloproteases (MMP) Gene Expression Profile and Their Diagnostic/Prognostic Potential. *BMC Cancer* **2019**, *19*, 581, doi:10.1186/s12885-019-5768-0.
153. Rodrigues, R.F.; Roque, L.; Rosa-Santos, J.; Cid, O.; Soares, J. Chromosomal Imbalances Associated with Anaplastic Transformation of Follicular Thyroid Carcinomas. *Br J Cancer* **2004**, *90*, 492–496, doi:10.1038/sj.bjc.6601530.
154. Bialek, J.; Kunanuvat, U.; Hombach-Klonisch, S.; Spens, A.; Stetefeld, J.; Sunley, K.; Lippert, D.; Wilkins, J.A.; Hoang-Vu, C.; Klonisch, T. Relaxin Enhances the Collagenolytic Activity and *In Vitro* Invasiveness by Upregulating Matrix Metalloproteinases in Human Thyroid Carcinoma Cells. *Molecular Cancer Research* **2011**, *9*, 673–687, doi:10.1158/1541-7786.MCR-10-0411.
